# Antithyroid drug induced agranulocytosis: what still we need to learn ?

**DOI:** 10.11604/pamj.2016.23.27.8365

**Published:** 2016-02-04

**Authors:** Liaqat Ali Chaudhry, Khalid Fouad Mazen, Ebtesam Ba-Essa, Asirvatham Alwin Robert

**Affiliations:** 1 Department of Internal Medicine, Pulmonary Division, Sultan Bin Abdulaziz Humanitarian City, Riyadh, Saudi Arabia; 2 Department of Anesthesia & Intensive Care Unit, Sultan Bin Abdulaziz Humanitarian City, Riyadh, Saudi Arabia; 3 Department of Medicine & Endocrinology, Dammam Medical Complex (MOH), Saudi Arabia; 4 Department of Endocrinology and Diabetes, Diabetes Treatment Center, Prince Sultan Military Medical City, Riyadh, Saudi Arabia

**Keywords:** Hyperthyroidism, ATD-antithyroid drugs, agranulocytosis, granulocyte colony stimulating factor

## Abstract

Antithyroid drugs (ATDs) induced agranulocytosis is a rare but life threatening condition. We report a 29 years Filipino female diagnosed as having hyperthyroidism with normal base line blood counts, liver and renal profile. She was started on maximum 60mg (20mg TID) oral dose of carbimazole since one month by her treating physician. Exactly after one month of treatment she presented to emergency room (ER) with fever, sore throat and generalized weakness for several days.

## Introduction

Thyrotoxicosis is a common (2% prevalence), endocrine disorder among the females of reproductive age. The first line drugs used for its treatment are carbimazole and propylthiouracil, where carbimazole is the commonest used medication since 1991 [1]. ATDs known as thiomides, containing sulfhydral group and coupled with thiourea component as Propylthiouracil (6-propyl-2-thiouracil) and methimazole (1-methyl-mercaptimidazole) are the most common drugs used in United States. Methimazole is used in Asia and Europe including Great Britain usually for 18 months. These agents are actively concentrated against molar concentration gradient. They act by inhibiting the thyroid peroxidase enzyme decreasing iodization, coupling of iodide and tyrosine aminoacids present at the thyroglobulin molecule consequently decreasing production of thyroxin and tri-iodothyronine [2]. Being main stay of treatment antithyroid drugs however is recognized cause of hematopoeitic damage including agranulocytosis which is rare (0.3-0.6%) but still a life threatening complication with a very high mortality (21.5%). High doses of ATDs are reported associated with increased incidence of agranulocytosis [3]. We present a patient who developed agranaulocytosis at one month of treatment on maximum dose of tablet carbimazole 20mg PO TID. She presented as having fever, sore throat and generalized weakness since 3 days and visited emergency room (ER) on three consecutive days. Temperatures recorded were 37.3c on first visit, 37c on 2^nd^ visit and, 38.5c during 3^rd^ visit respectively with low white blood count done consistent with agranulocytosis.

## Patient and observation

29 years of old married Filipino female working as baby care presented on 25-02-2015 to medical outpatient department (OPD), with progressive swelling in front of her neck and heat intolerance since few months with irregular cycles. She is married since 3 years living with her husband but no history of conception. She had no past history of relevance. She was diagnosed on 19-3-2015 in medical OPD as having severe hyperthyroidism based on her thyroid functions reports. Her thyroid hormone assay at the time of diagnosis was Thyroid stimulating hormone (TSH) = 0.0005Miu/L, free thyroxine (FT4) = 73.6pmol/L and free tri-iodothyroxine (FT3) = 46.08 pmol/L respectively. Her base line complete blood counts on 19-03-2015 were, WBC = 8.0X10^6/micL, Neutrophils = 52%, Lymphocytes = 41%, Monocytes = 0%, Basophils = 2%, Eosinophils = 1%, Red blood cells = 4.6x10^6/micL, Hemoglobin = 14gm/dL, Platelets = 284x10^3/micL, random blood glucose = 143mg/dL, renal and liver profile being normal, pregnancy test negative, Chest x-ray and ECG being unremarkable. Her blood pressure = 117/78 mmHg, Heart rate = 90/min, Respiratory rate =18/min, and oxygen saturation (O2 SAT %) at room air were 99% respectively. She was afebrile and her booking weight was 57kgs. She was started on oral carbimazole (neomercazole) 20mg PO TID for one month along with Tab. Inderal 10mg PO TID with a one month supply of medications. Her next appointment was fixed after one month by the treating physician and there was no documentation on the possible drug related adverse effects.

On **31-03-2015** she first time came to hospital emergency room (ER) complaining having generalized prostration and fever, her vitals were Blood Pressure (BP) = 110/70mmHg, heart rate (HR) = 88/min, respiratory rate (RR) = 19/min, oxygen saturation (O2Sa) t = 97% at room air, Temperature = 37.3c. She was reassured by the ER physician and discharged with Tab. paracetamol 1gm PO TID PRN. She was not asked about current medications and she did not disclose either. Before coming to ER she had taken 2 tablets (1gm) of paracetamol including her routine dose of carbimazole and Inderal 10 mg, and continued taking antithyroid medications. During her 2nd visit to the ER on 12-04-2015, with the same complaints, her vitals were unremarkable and her temperature was normal at 37°C, because even this time, she took as usual paracetamol and her dose of carbimazole including 10 mg inderal tablet before visiting the ER. She was reassured by the attending ER physician and discharged again; no laboratory work up was advised. On 16-04-2015 during her 3^rd^ visit to ER, she was referred to me as back-up on call physician by the ER physician for having unexplained low blood counts, fever at 38.5°C, sore throat, prostration, BP = 110/70 mmHg, Heart rate = 112/min regular, Respiratory rate = 20/min, O2Sat = 99% at room air, weight = 57 KG, and has a rash like face as noted by the ER physician.

**Figure 1:**
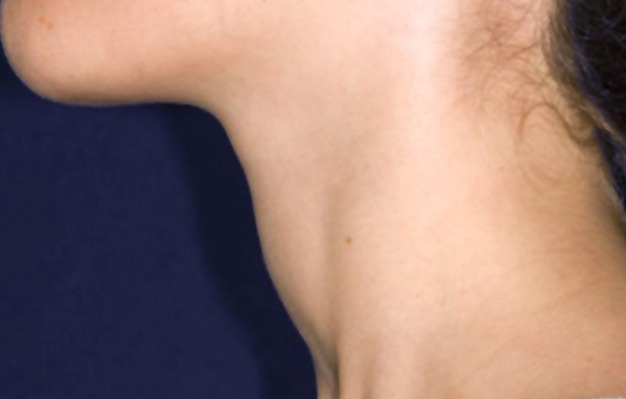
Goitre (hyperthyroidism)

This young female Filipino (Muslim) was sitting on the edge of the bed covering her head, neck and upper torso with her hijab. Visible part of her face was blushed red manifesting hyperdynamic circulation but there was no specific skin rash otherwise. While asking her current medications on repeated questions about antibiotics or other drugs, at last only then she disclosed that she is taking treatment for her thyroid in the form of carbimazole, the dose of which she had taken even today just before coming to the ER. In addition when she was asked previously you were not having temperature when examined in ER she disclosed that usually before coming to the ER during her earlier visits she used to take 2 tablets of paracetamol. On removing the hijab, an obvious diffuse goiter was visible (Figure 1), rest of her systemic examination was unremarkable, heart rate = 112 and regular. In the light of history of her current antithyroid medications and low white blood counts WBC = 1.11X10^3/micL, Neutrophil = 11%, Lymphocytes = 78%, Hemoglobin = 13.4gm/dL, red blood cells (RBC) = 4.3X10^6/micL, Basophils = 1%, Monocytes = 0%, Eosinophils = 10%, Platelets = 279x10^3/micL random blood glucose =143mg/dL, a diagnosis of ATD induced agranulocytosis was made. Her ATDs were discontinued and was admitted to medical HDU (high demand unit) under reverse isolation. After sending her septic workup, was started on injection Meropenem 1gm iv q8 hrs, ictaconazole 100mg PO OD, Tab. paracetamol 1gm po q8hrs prn, 5%DW/N. saline IV 50ml/hr. Inderal 10mg PO TID was continued, Granulocyte colony stimulating factor (GCSF) as filgrastim, in a dose of 300micrograms SC OD was requested, which was difficult to arrange. Her complete blood counts from baseline to day of discharge are shown in Table 1. On 2^nd^ day of admission 17-4-2015 she had highest temperature 40c, her BP = 90/60mmHg, heart rate = 122/min, Respiratory rate = 22/min, 02 SAT = 100% at room air, she vomited twice and had one loose motion. Her WBC reported= 3.25x10^6/micL, Neutrophils = 1%, Lymphocytes = 93%, Monocytes = 2%, Basophils = 0%, Eosinophls = 9%, Patelets = 224x10^3/micL, RBC= 4.5X10^5/micL. Being skeptical of this complete blood count report, still she received first dose 300 mcg SC of GCSF in the afternoon. Same antibiotics were continued, Inderal was discontinued in regular doses in the anticipation of septicemia or septicemia shock, as her blood pressure was on the lower side with hyperpyrexia.

**Table 1:** Complete blood counts baseline and admission to the day of discharge

Days	WBC X10^6/μL	RBC 5E+6 cells/μL	PlateletsX10^3/μL	Neutrophil (%)	Lymphocytes (%)	Monocytes (%)	Basophil (%)	Eosinophil (%)
Baseline 19-03-2015	8	4.6	284	52	41	0	2	1
After one month treatment with Antithyroid drug admitted via emergency room with fever and sore throat								
Day 1 16-04-2015 (HDU)	1.11	4.3	279	11	78	0	1	10
Day 2 17-04-2015*	3.25	4.5	224	1	93	2	0	9
Day 3 18-04-2015	1.07	4.3	237	1	91	5	0	4
Day 4 19-04-2015	2.02	4.2	300	1	94	4	0	1
Day 5 120-04-2015	0.84	4.5	370	1	95	5	1	7
Day 6 21-04-2015	0.54	4	265	1	92	7	0	4
Day 7** 22-04-2015	0.65	5	400	1	92	8	0	4
Day 8*** 23-04-2015	0.75	4.7	288	1	92	10	0	3
Day 9 24-04-2015	0.67	4.7	275	1	88	10	0	1
Day 10 25-04-2015	0.59	5.2	255	4	85	11	0	0
Day 11 26-04-2015	0.77	4.7	277	28	56	14	1	1
Day 12 27-04-2015	1.73	4.3	278	38	45	15	2	0
Day 13 28-04-2015 Discharged	2.16	4.7	300	64	23	10	2	1

Day 3^rd^ 18-4-2015, WBC = 1.07x10^6/micL, Neutrophil count = 1%, Lymphocytes = 91%, Mnocytes = 5%, Eosinophils = 4%, Basophils = 0%, Platelets = 237X10^3/micL, HB = 13.2, RBC = 4.3, BP = 100/5 8 mmHg, HR= 110/min, RR= 20/min, O2SAT = 99% at room air, Temp = 38.5c, liver function tests (LFTs) and renal function tests (RFTs) were normal, bowels and urinary habits normal and no more loose motions and vomiting. 4^th^ day 19-4-2015, She was still febrile, Temp = 38c and WBC = 2.02x10^6/micL, neutrophils = 1%, Lymphocytes = 94%, monocytes = 4%, Basophils = 0%, Eosinophils = 1%, RBCs and platelets being normal. Keeping in view the possibility of pseudomonas aeroginosa which is a common pathogen in agranulocytosis, and not strongly covered by meropenem, antibiotic treatment was changed to injection ceftazidime 1gm IV TID, while rest of the management continued as before. Urine bacterial culture reported negative. Thyroid functions reported, TSH = 0.0006mIU/L, FT4 = 26.21pmol/L, FT3= 9.63Ppmol/L respectively, this was after about one month of treatment with carbimazole 20mg Po TID. Day 5^th^ 20-4-2015, the patient was feeling better and her BP 110/75 mmHg, HR = 90/min, RR = 19/min, O2SAT = 99% at room air, Temp = 37.4°C, WBC= 0.84x10^6/micL, Neutrophil = 1, Lymphocyte = 95%, Mononcytes = 5%, Basophils = 1%, Eosinophils = 7%, she was feeling warm, she was still very particular with her hijab, she was asked to remove the upper torso covers and use light weight hijab. Throat swab culture reported klebsiella pneumonia sensitive to meropenem and ceftazidime, ceftriaxone. Same antibiotic was continued and her Inderal was restored 10mg PO TID, because now her BP was acceptable = 110/70 mmHg. Day 6^th^ 21-4-2015, She was feeling much more better no more fever, eating well and asking to be discharged, having BP = 115/70mmHg, HR = 100/min, RR = 19/min, O2 SAT = 99%, Temp = 37°C. Her stool and blood culture reported negative. Her WBC = 0.54x10^6/micL, Neutrophil = 1%, Lymphocytes = 92%, Monocytes = 7%, Eosinophils = 4%, Basophils = 0%. Granulocyte colony stimulating factor (GCSF) filgrastim was not available. An external hematology consultation was done at this stage. Day 7^th^ 22-4-2015, she was feeling warm but having normal temperature round the clock, BP = 115/75mmHg, HR = 104/min regular, RR = 19/min, O2 SAT = 99%. WBC = 0.65x10^6/micL, Neutrophils = 1%, Lymphocytes = 92%, Methicillin resistance Staphylococcus aureus (MRSA) screening reported negative. At this stage control of her thyroxine hormone related effects was considered and her treatment was revised with addition of 5% Lugol's iodine solution 2 drops PO TID which unfortunately was not available, her dose of Inderal was increased to 20mg PO TID, and she was started on injection dexamethasone 4mg IV q8H, while empirical antibiotic inj. ceftazidime 1gm IV was continued to cover pseudomonas aeroginosa the commonest organism in agranulocytosis. She received 2nd dose of GCSF 300mcg arranged from other hospital pharmacy. Hematologist advised to continue the same treatment comprised of corticosteroids, GCSF along with antibiotics, but advised bone marrow biopsy as well. Day 8^th^ 23-4-2015, same line of management was continued without any change, and bone marrow biopsy was deferred till 4 weeks. She remained stable and afebrile, insisting for discharge, BP = 115/75mmH, HR = 84/min, RR = 19/min, O2SAT = 99% at room air, WBC = 0.75x10^6/micL, Neutrophil = 1%, Lymphocytes = 92%, Monocytes = 10%, Basophils = 0%, Eosinophils = 3%, she received 3rd and the final dose of 300mcg SC of GCSF, as it was no more available.

Day 9^th^ 24-4-2015, she was asymptomatic and stable vitals, BP115/75mmHg, HR = 84/min, RR = 18/min, O2SAT = 99%, afebrile. Her WBC = 0.67x10^6/micL, Neutrophil = 1%, Lymphocyte = 88%, Monocytes = 10%, Basop-hils = 0%, Eosinophils = 1%, normal range platelets and RBCs. Day 10^th^ 25-4-2015, She was asymptomatic, and her WBC = 0.59x10^6/micL, Neutrophil = 4%, Lymphocytes = 85%, Monocytes = 11%, Eosinophils = 0%, Basophils = 0%. Today first time complete blood counts showed beginning of neutrophil count rising with normal RBCs and platelets. Her heart rate was in normal range. Day 11^th^ 26-4-2015 and day 12^th^ 27-4-2015, her full blood counts were WBC = 0.77x10^6/micL, Neutrophil = 28%, Lymphocytes = 56%, Monocytes = 14%,Eosinophils = 1%,Basophils = 1% and WBC = 1.73x10^6/micL, Neutrophil = 38%, Lymphocytes = 45%, Monocytes = 15%, Basophils = 2%, Eosinophils = 0% respectively. As she had only two options for radical treatment: either surgery or radioactive iodine. A surgical consultation was done. She refused both the options despite being educated about the adverse effects of antithyroid medications and different available treatment options. Day13^th^ 28-4-2015, she remained asymptomatic and was having normal vital signs. Her blood counts reported, WBC = 2.16x10^6/micL, Neutrophils = 64%, Lymphocytes = 23%, Monocytes = 10%, Eosinophils = 1%, Basophils = 2%, Platelets and RBCs being in normal range. She was discharged on Ciprofloxacin 500mg PO bid for 5days, oral Prednisolone in tapering dose, tablet Inderal 30mg PO TID, tablet Omeprazole 20mg PO OD and was referred to the endocrinologist in an acute care hospital for further advice.

## Discussion

Hyperthyroidism is a common endocrine condition more common in females in their reproductive years and main stay of treatment has been antithyroid drugs (ATD) since 1941[4]. Surgery and radio-iodine were the other options. Carbimazole and thiouracil are the commonest antithyroid drugs used, and both could equally cause agranulocytosis or pancytopenia. Whereas hyperthyroidism or thyrotoxicosis is mostly a benign condition provided diagnosed early, its treatment with antithyroid drugs could cost the life of the patient causing fatal complications like agranulocytosis (0.3-0.6%) or pancytopenia. Factors indicating bad prognosis in ATDs induced hematopoietic damage are: age >65 years, pancytopenia, co-existing liver and renal insufficiency. Patients may not have any symptoms or vague complaints and early clinical diagnosis become difficult under these circumstances. It is therefore mandatory to ask complete blood counts and liver functions at specific time i.e at 2 weeks of ATD treatment to guard against ATDs induced agranulocytosis in asymptomatic patients [4, 5]. Diagnosis of agranulocytosis is made when patients show a granulocyte count below 5 > 00 /ul after ATD therapy [5]. ATD induced pancytopenia is defined when in addition to agranulocytosis patients show hemoglobin less than 11gm/dl and a platelet count less than 100x10/liter. Recovery from ATD induced agranulocytosis or pancytopenia is defined as improvement in granulocyte counts to 500/ul or more. Hematopoetic damage could be present without patients manifesting any symptoms, therefore asking a full blood count at every 2 weeks is recommended [6]. Patients’ needs to be educated by the clinicians with full documentation about signs and symptoms of ATD induced side effects like fever, sore throat, prostration,skin rash etc, where they should be asked to stop taking medications and seek medical advice. Who will develop hematopoetic damage is not pre-determined despite of various postulated underlying mechanisms mentioned below: a)Antibodies against antithyroid drug bound to the wall of the granulocyte cause accelerated destruction of the granulocytes; b)Antibodies may target drug/metabolite complex adsorbed to the neutrophil in the presence of a plasma /complement component; c)The drug may trigger the production of autoantibodies targeting granulocytes or drug metabolites may directly target neutrophils when used in higher doses; d)Interaction of a granulocyte antigen and drug may induce the production of antibodies; e)Besides above mechanisms, idiosyncratic drug induced agranulocytosis (which is independent of the dose) is a severe selective depression of myelopoeisis. It is due to an unpredictable adverse reaction to a wide variety of drugs in hypersensitive individuals-occurring in first 1-2 months of taking antithyroid drug. In the end it could just be labelled as a random bad luck [7].

ATD induced agranulocytosis when present, is 10 times more common than pancytopenia. Mortality in agranulocytosis is high up to 21.5% and much higher in pancytopenia. Overwhelming infections are the cause of life threatening septicemia and septicemic shock. ATD induced hematopoetic damage usually is anticipated after 2 weeks to 14 weeks. Fever and sore throat were the most common presenting complaints besides prostration. Besides complete blood counts liver function also needs to be monitored simultaneously [8]. Renal and liver functions remained normal in our patient. Patients are usually treated conservatively with supportive measures after stopping the offending drug. Patients are kept in reverse isolation and after sending the septic lab work up, are started emperical broadspectrum antibiotics for the most commonly expected bacteria like streptococcus pneumoniae, klebsiella pneumoniae, *Pseudomonas aeruginosa* etc, iv fluids, antifungal medications. The use of GCSF since 1991, has improved the prognosis by reducing the recovery time of the granulocytes in large majority of the patients, but despite of its use, mortality still remains high up to 5% [9]. GCSF is less effective in ATDs induced pancytopenic patients, but still is more effective here compared to bone marrow failure induced by cancer therapy or radiations. Bone marrow biopsy is better deferred till four weeks; it takes about 5 days to 2 weeks for the neutrophil recovery. GCSF better be continued till desired response. Simultaneous addition of corticosteroids like dexamethasone 4 mg iv q8h or methylprednisolone 1gm IV q8h has augumenting anti-inflamtory effect on recovery of the hematopoietic stem cells [10]. The most common and better tolerated daily dose of Carbimazole used is in range of 15-45mg given in three divided doses. Supply of ATDs is selectively given according to the reliable intelligence and knowledge of the patient. It is safe to supply 2 weeks treatment to those less educated as our patient; she went on taking ATD despite of being sick. Maximum or higher doses at the start better be avoided. At 4 weeks the dosage could be safely revised up or down according to the response [11]. Patients’ needs to be educated by the clinicians about signs and symptoms of ATD induced side effects with full documentation. None of the two were done in this case as revealed in the medical records. Adverse effects include fever, sore throat, prostration, skin rashes, liver derangement where patients are asked to stop taking medications and seek immediate medical advice. All patients have not the same knowledge and understanding about the adverse effects of ATDs. The subject patient was started on the maximum dose of ATDs from the start and at the top of that was given supply for the whole one month which she continued taking even after being sick. It is therefore safe to prescribe 2 weeks supply of ATDs in the start, especially to less educated patients as ours. Bone marrow in cases of antithyroid induced hematopoetic depression is non-contributory at least initially, and better be deferred till 4 weeks. It is because, after stopping the offending drug (ATD), starting broad spectrum antibiotics and giving GCSF continuously with concomitant corticosteroids till neutrophilic response, has really improved the outcomes in large majority of patients. Despite of all these measures still there is 5% mortality rate reported, therefore prevention and early detection of antithyroid induced agranulocytosis has prognostic importance.

## Conclusion

Severity of hyperthyroidism and doses of ATDs better be judged and decided based on symptoms and not on the figures (values) of the thyroid hormones. All patients have not the same knowledge and understanding about the adverse effects of ATDs. It is therefore safe to prescribe 2 weeks supply of ATDs in the start, especially to less educated patients as ours. The initiation of carbimazole in average dose range of 15-45 mg in divided doses and supply of 2 weeks medication is a safe choice. After 4 weeks antithyroid treatment, the dosage could be revised according to the response. Patients’ needs to be educated by the clinicians about signs and symptoms of ATD induced side effects with full documentation. Conducting routine complete blood counts at 2 weeks on wards is beneficial in alerting the clinicians to the possibility of ATD induced agranulocytosis, it may detect those cases having no symptoms but still having low blood counts predicting underlying pre-disposition to hematopoetic damage. History of current medications needs to be given due importance. This patient was diagnosed having ATD induced agranulocytosis during her 3rd ER visit, by virtue of taking history of current medications coupled with her low WBC and neutrophil counts. Availability of Broad spectrum antibiotics has improved the prognosis and outcomes of ATD induced agranulocytosis. Further improvement in prognosis is attributed to the use of GCSF by decreasing the duration of neutrophil recovery in ATD induced agranulocytosis. But despite of GCSF utility, mortality remains high (5%), especially in pancytopenic patients, therefore its prevention and early detection has prognostic value. Bone marrow is non- contributory in ATDs induced hematopoetic injury at least initially and better be deferred until 4 weeks. It is because,after stopping the offending drug (ATD), starting broad spectrum antibiotics and giving GCSF continuously with concomitant corticosteroids till neutrophilic response, has really improved the outcomes in large majority of patients. On the contrary bone marrow suppression induced by chemotherapy or radiations has guarded prognosis and takes longer time to respond if at all. It is here when an early bone marrow biopsy may help to tailor treatment and predict prognosis.
