# Erratum: antithyroid drug induced agranulocytosis: what still we need to learn?

**DOI:** 10.11604/pamj.2016.24.250.10044

**Published:** 2016-07-18

**Authors:** The Pan African Medical Journal

**Affiliations:** 1 The Pan African Medical Journal

**Keywords:** PAMJ, erratum, agranulocytosis

## Abstract

This erratum corrects article: ″ Antithyroid drug induced agranulocytosis: what still we need to learn ″ The Pan African Medical Journal. 2016;23:27. doi:10.11604/pamj.2016.23.27.8365

## Erratum

The original version of this article [1] contained typographical errors in its title and author names. The word ″agranulocytosis″and the name of one of the authors were misspelt. The author's name has been changed to Khalid Fouad Mazen. These have been corrected in both the PDF and HTML versions of the article.
